# Utilization of Oral Healthcare Among Uninsured Populations During the COVID-19 Pandemic in the United States

**DOI:** 10.7759/cureus.75609

**Published:** 2024-12-12

**Authors:** Emily Singer, Samin Panahi, Brenda Spearman, Akiko Kamimura

**Affiliations:** 1 Family and Consumer Studies, The University of Utah, Salt Lake City, USA; 2 Epidemiology, The University of Utah, Salt Lake City, USA; 3 Management, Maliheh Free Clinic, Salt Lake City, USA; 4 Sociology, The University of Utah, Salt Lake City, USA

**Keywords:** covid-19, free clinics, oral health, uninsured, utilization

## Abstract

Background: Accessibility to dental care is vital for uninsured low-income individuals. There is a deficit of research that examines oral healthcare seeking during the COVID-19 pandemic among uninsured low-income individuals. The purpose of this study is to describe oral health-related issues among patients of a free clinic that does not provide dental care during the COVID-19 pandemic.

Methods: The sample consists of 254 patients of a free clinic located in the Intermountain West. Data were collected by distributing self-administered paper surveys to patients 18 or older who spoke English and/or Spanish from December 2021 to May 2022.

Results: Nearly 40% of study participants reported being long overdue for dental appointments, with many not receiving care within the past two years (n = 96, 37.8%). Less than 30% of free clinic patients had a dental appointment within the past six months (n = 73, 28.9%). Cost is the most substantial barrier for free clinic patients to receive dental care (n = 150, 60%).

Conclusion: This study's findings bridge the research gap on dental care experience during the pandemic among uninsured populations and are helpful in increasing knowledge for improving access to dental care at free clinics.

## Introduction

In the United States (U.S.), access to oral health care is greatly determined by socioeconomic status [1]. Poor oral health is most common among people who are low-income, uninsured, and/or minority races/ethnicities, immigrants, or rural populations [2]. Insurance coverage of dental care is another barrier to access to care; those with dental insurance visit the dentist at twice the rate of those without dental insurance [3]. Limited access to obtaining dental care compromises oral health.

Without adequate access to dental care, biannual visits to the dentist to maintain optimal oral health are unobtainable for at-risk populations. Preventative care and early disease detection are vital. Without regular dental checkups, individuals experience cavities, dental caries, oral pain, and permanent tooth decay at increased rates [4]. Poor oral health is a predisposition for poor systemic health. Atherosclerotic disease, pulmonary disease, diabetes mellitus, pregnancy complications, and low birth rate are examples of diseases that can be induced as a direct result of poor oral health [5]. Oral health complications and subsequent systemic complications can be avoided or caught early with consistent preventative care.

On March 11, 2020, the World Health Organization declared COVID-19 a global pandemic, with more than 118,000 cases in 114 countries, which was a 13-fold case increase in two weeks [6]. The American Dental Association advised dentists to postpone all nonurgent/emergency procedures on March 16, 2020 [7]. The COVID-19 pandemic amplified preexisting socioeconomic barriers to accessing healthcare. Underserved communities encountered heightened challenges due to the pandemic in meeting their oral health needs and concerns [8].

Public health preventative dental care programs have been severely limited with the arrival of the COVID-19 pandemic [9]. Government-issued school closures halted school dental programs, which provided oral healthcare for children who would not otherwise have access [10]. Low-income families greatly benefit from these programs, as they are low-cost and have a significant impact [9]. Other factors impacting access to oral healthcare during the pandemic are unemployment and lack of insurance. The pandemic brought about an unprecedented rise in unemployment; on April 4, 2020, over 16.5 million initial unemployment claims were reported, with six to seven million new claims arriving weekly [11]. Many people of lower socioeconomic status with entry-level jobs found themselves out of work due to the pandemic [12]. Unemployment often jeopardizes insurance benefits, making people less likely to have dental insurance and less likely to seek care [13]. Providing dental services at safety-net healthcare facilities, such as free clinics, is extremely important so that uninsured individuals have access to care.

It is important to examine the extent to which the pandemic has affected the oral care of underserved populations. This study aims to describe the oral health habits of underserved populations during the pandemic. In particular, the participants were classified for analysis based on language and nativity because previous studies with the clinic showed that patients in each language/nativity category were very different from each other in terms of health outcomes and health-related behaviors. This research aims to fill the knowledge gap of the pandemic’s impact on the underserved.

## Materials and methods

Setting

The study population comprises Maliheh Free Clinic patients in Salt Lake City, UT. The clinic is a nonprofit organization, open all weekdays, and provides same-day appointments for urgent medical conditions, adult family medicine, pediatric care, assistance in obtaining medications, adult and childhood immunizations, on-site laboratory testing, X-rays, ultrasounds, screening mammograms, women’s health, diabetes specialty clinic, and healthy living classes. The clinic does not provide dental care, emergency care, prenatal care, and prescriptions for controlled substances due to its limited resources. The clinic offers care to uninsured individuals whose household income level is 200% under the federal poverty level. For a family of four people, the total annual income must not exceed $55,500 to qualify for care in 2022. Patients must present proof of income in the form of the most recent tax return, three most recent paycheck stubs, the last three months of bank statements, or a letter from an employer or religious leader to be eligible to use the clinic’s services. The clinic primarily serves individuals aged 18-64 and largely Hispanic patients. Because the clinic is not an urgent care, patients typically form a relationship involving continuity of care with the clinic.

Data collection and participants

This study was approved by the University of Utah’s Institutional Review Board (00072275). This is a cross-sectional study utilizing a self-administered paper survey. Data were collected based on convenience sampling in the waiting room of the clinic from patients who met the qualifications of being 18 years or older, were able to read English or Spanish, and had current patient status at the time of their appointment From October 21, 2021, to May 18, 2022. A research team member was present in the clinic lobby to facilitate the distribution and collection of surveys and was available to answer questions. Participants were presented with paper versions of the survey in English and Spanish. After consenting to participate in the study from each participant, clinic patients completed the survey while waiting for their appointment to begin.

Measures

Questions pertinent to this study consist of topics about demographics, COVID-19, and oral health.

Demographics

Information on participants’ age, gender, nativity, country of origin, race/ethnicity, level of education, employment status, and martial/relationship status was collected. Participants were also asked to describe their overall health by choosing either “Excellent,” “Very good,” “Good,” “Fair,” or “Poor.”

COVID-19

Following demographics, COVID-19-related questions asked whether the participant had tested positive if they tested positive and were hospitalized, and if they had a family member in the same household who tested positive. Participants were also asked to compare their current to prepandemic general health.

Oral health

Oral health-related questions were extracted from the 2011-2012 National Health and Nutrition Examination Survey [14]. Participants selected the time frame in which they last visited the dentist, including dental hygienists, orthodontists, oral surgeons, and all other dental specialties. Participants were then asked if there was a time during the past 12 months when they needed dental care but were unable to get it at the time. If they were unable to get care, participants were also asked the reason(s). The response options were: “Could not afford the cost,” “Did not want to spend the money insurance did not cover,” “Recommended procedures,” “Dental office is too far away,” “Dental office is not open at convenient times,” “Another dentist recommended not doing it,” “Afraid or do not like dentists,” “Unable to take time off from work,” “Too busy,” “I did not think anything serious was wrong or expected dental problems to go away,” “Other,” and “Not applicable (I could get needed dental care).”

Data analysis

Participants’ survey responses were recorded and cleaned on an Excel spreadsheet (Microsoft Corporation, Redmond, WA) and then analyzed using Statistical Package for the Social Sciences version 28 (IBM Corp., Armonk, NY). Descriptive statistics and regression analyses were performed to describe variables and examine factors associated with oral health behaviors during the pandemic.

## Results

Table 1 describes the sociodemographic characteristics of study participants (n = 254). The participants were classified based on language and nativity because previous studies with the clinic show that patients in each language/nativity category were very different from each other in terms of health outcomes and health-related behaviors. The study population comprises 169 (66.5%) female and 89 (33.5%) male participants, with an overall mean age of 43.95. Fourteen (5.5%) participants are Asian or Pacific Islander, 27 (10.7%) participants are White/Non-Hispanic, and 206 (81.4%) are Hispanic/Latino/Latina participants. More than half of the participants are Spanish speaking (n = 150, 59.1%), with 148 (98.7%) being Hispanic/Latino/Latina Spanish speakers. Approximately half (n = 128, 50.4%) of the sample indicated they were currently employed. Slightly more than one-third (n = 96, 37.8%) of participants have been patients of the free clinic for more than two years.

**Table 1:** Sociodemographic characteristics of participants and descriptive statistics The values are represented as frequency (%) p value denotes significance from Pearson’s chi-square tests comparing U.S.-born English speakers, non-U.S.-born English speakers, and Spanish speakers N.S.: not significant; N/A: not applicable (due to a cell less than n = 5)

Variable	Total sample (n = 254)	U.S.-born English speakers (n = 36)	Non-U.S.-born English speakers (n = 68)	Spanish speakers (n = 150)	p value
Female	169 (66.5)	18 (50.0)	45 (66.2)	106 (70.7)	N.S.
Race/ethnicity					
White/Non-Hispanic	27 (10.7)	21 (60.0)	5 (7.4)	1 (0.7)	N/A
Hispanic/Latino/Latina	206 (81.4)	11 (31.4)	47 (69.1)	148 (98.7)	<0.01
Asian or Pacific Islander	14 (5.5)	2 (5.7)	12 (17.6)	0	N/A
Some college or higher	118 (46.5)	20 (55.6)	33 (48.5)	65 (43.3)	N.S.
Currently employed	128 (50.4)	19 (52.8)	40 (58.8)	69 (46.0)	N.S.
Currently married	103 (40.6)	7 (19.4)	27 (39.7)	69 (46.0)	<0.05
Patient of the clinic: two years or longer	98 (38.6)	12 (33.3)	32 (47.1)	54 (36.0)	N.S.
Had tested positive for COVID-19	85 (33.6)	6 (17.1)	20 (29.4)	59 (39.3)	<0.05
Family tested positive for COVID-19	90 (36.0)	9 (26.5)	19 (28.4)	62 (41.6)	N.S.
Time since last dental visit					
6 months or less	73 (28.9)	10 (27.8)	23 (34.3)	40 (26.7)	N.S.
6 months to 1 year	45 (17.8)	3 (8.3)	13 (19.4)	29 (19.3)	N.S.
1-2 years	39 (15.4)	5 (13.9)	8 (11.9)	26 (17.3)	N.S.
More than 2 years	96 (37.8)	18 (50)	23 (33.8)	55 (36.7)	N.S.
Main reason of the last dental visit (top two answers)					
Check-up/examination/cleaning	120 (48.6)	13 (36.1)	40 (59.7)	67 (46.5)	N.S.
Something wrong/bothering/hurting	15 (6.1)	3 (8.3)	3 (4.5)	9 (6.3)	N/A
Could not receive needed dental care (past 12 months)	141 (56)	27 (75)	29 (43.3)	85 (57)	<0.01
Could not get needed dental care due to cost	150 (60)	24 (68.6)	39 (58.2)	87 (58.8)	N.S.

The overall mean age was 43.95 (SD = 15.58) (Table 2).

**Table 2:** Age of participants The values are represented as mean ± SD p < 0.01 denotes significance from ANOVA tests for continuous variables comparing U.S.-born English speakers, non-U.S.-born English speakers, and Spanish speakers SD: standard deviation; ANOVA: analysis of variance

Total sample (n = 254)	U.S.-born English speakers (n = 36)	Non-U.S.-born English speakers (n = 68)	Spanish speakers (n = 150)	F Statistic
43.95 ± 15.58	36.36 ± 15.41	43.59 ± 16.24	45.95 ± 15.58	5.73

COVID-19

The overall frequency of reported positive COVID-19 tests is 85 (33.6%). Of 150 Spanish speakers, 59 (39.3%) tested positive for COVID-19, and of 36 U.S.-born English speakers, six (17.1%) tested positive for COVID-19. In addition, 62 (41.6%) of Spanish speakers and nine (26.5%) U.S.-born English speakers had family members who tested positive for COVID-19.

Oral health

When asked about the amount of time since the participants’ last dental visit, the most frequent response of the total study sample was more than two years (n = 96, 37.8%). Visiting the dentist more than two years ago was the most frequent response of U.S.-born English speakers (n = 18, 50%) and secondly Spanish speakers (n = 55, 36.7%). Among the total sample population, a visit to the dentist six months or less was the second most frequent response, with 28.9% (n = 10) for U.S.-born English speakers and 26.7% (n = 40) for Spanish speakers. The main reason for participants’ last dental visit was for checkup/examination/cleaning (n = 120, 48.6%). A total of 150 (60%) of the sample participants could not get the dental care they needed due to cost. Cost-prohibitive reasons for not getting needed dental care are applicable to 24 (68.6%) U.S.-born English speakers, 39 (58.2%) non-U.S.-born English speakers, and 87 (58.8%) Spanish speakers.

## Discussion

This project explored dental care during the COVID-19 pandemic and has three main findings. First, most free clinic patients’ last dental visit was over two years ago. Second, not many responders had a dental visit within six months at the time they took the survey. Third, more than half of free clinic patients could not get the care they needed due to cost. As demonstrated by this study, free clinic patients face setbacks in obtaining optimal oral health.

It has been at least two years for many free clinic patients since having a dental appointment. Dental visits are often difficult for uninsured individuals to come by, especially with the added factor of the COVID-19 pandemic. Before the pandemic, people of self-rated higher socioeconomic status populations faced fewer difficulties in seeking dental visits than those of self-rated lower socioeconomic status [15,16], and the difference in accessibility to care was exacerbated during the pandemic. This research study adds data on oral health among individuals of low socioeconomic status. Data collection took place approximately a year and a half to two years after the World Health Organization’s official declaration of the COVID-19 pandemic. There is a possibility that the COVID-19 pandemic amplified preexisting health inequalities [17-19], which directly correlates to the increase in free clinic patients not seeking dental care in the past two years.

Since many free clinic patients did not have dental care within the past two years, it is logical to assume that the number of patients who sought treatment within the past six months is also low. The main reason study participants last visited the dentist was for a checkup, examination, or cleaning. Thus, these patients most likely went to the dentist for their recommended biannual visit in the past six months despite encountering possible difficulties accessing oral health care during the pandemic. A previous study with the same free clinic, conducted before the COVID-19 pandemic, reports that 18.3% of study participants received preventative care within the past six months [20]. Compared to the mid-pandemic number of patients who reportedly visited the dentist in the past six months, the prepandemic study results indicate a slightly higher percentage of patients who could receive care. This may be explained by the fact that the free clinic offered dental services at the time of the first study, and 9.9% of study participants received dental care at the clinic [20]. Even when the free clinic provided dental services, most patients were not able to receive their care, which is likely accredited to limited availability and variety of offerings.

The pandemic brought a rise in telehealth appointments, altering healthcare operations. While teledental appointments proved to be advantageous in some respects [21,22], virtual appointments cannot adequately replace dental checkups, examinations, and cleaning, which serve as invaluable measures of preventative care. The pandemic’s normative virtual dental appointments exacerbate yet another prepandemic discrepancy widespread among low-income populations: a lack of internet access [23]. A lack of preventative care and lack of access to the primary form of dental care being practiced during the COVID-19 pandemic are two added challenges to free clinic patients’ existing obstacles to maintaining optimal oral health.

Cost-prohibitive factors proved the main reason free clinic patients could not obtain needed dental care. Not all free clinics offer dental care as a service, so the cost, even before the pandemic, is a barrier to receiving care. Many vulnerable populations describe dental care as unaffordable [23-26]. Decreased access to the limited free dental care that existed before the pandemic likely added to participants’ inability to afford treatment. Additionally, pandemic-induced financial burdens disproportionally struck low-socioeconomic populations [27]. When finances run tight, dental care likely takes low priority. Consequently, being unable to obtain dental care due to cost is prominent among free clinic patients.

There are limitations to this study, as it is a small-scale project with only 254 participants. Data collection had limitations because, due to the pandemic, in-person appointments were reduced and replaced with telehealth appointments. This meant there were fewer potential study candidates at the clinic than there would have been prepandemic, yielding a small sample size. The online survey was not utilized because the percentage of free clinic patients who have internet access is low. An additional limitation is the lack of prepandemic data. Furthermore, since this study focused on describing, there is no strong theory behind it. Finally, this free clinic’s population is primarily Hispanic, which is not necessarily representative of all free clinic populations. The findings in this study may be most applicable to other free clinics with a majority of Hispanic patients but not free clinics with different dominant populations.

## Conclusions

This study brings forth findings on uninsured populations’ oral health amidst a global pandemic. The data found in this study demonstrate that the COVID-19 pandemic reduced access to oral health care, exacerbating prepandemic dental disparities among free clinic patients. Free clinic patients need extra support in obtaining dental care. Although teledental appointments can be useful for addressing patients’ minor dental concerns, there must be an alternative to traditional telehealth dental appointments for free clinic patients when in-person visits are unavailable, such as the provision of temporary free internet access from homes. Future research could include longitudinal studies at this same free clinic to track changes in patients’ oral health over time after the pandemic. Multisite research may also prove beneficial to collect data on different populations who may have different needs and barriers. More future research relating to this study could include qualitative studies involving patient interviews regarding their oral health habits to capture patient experiences in details. The findings would be helpful to better understand oral health disparities.

## Appendices

1. Compared to before the COVID-19 pandemic (before March 2020), how would you rate your health in general now?

a. Much better now than one year ago

b. Somewhat better now than one year ago

c. About the same

d. Somewhat worse now than one year ago

e. Much worse now than one year ago

2. Have you tested positive for COVID-19?

a. Yes

b. No

3. If you have been tested positive for COVID-19, were you hospitalized for COVID-19?

a. Yes, I was hospitalized

b. No, I was not hospitalized

c. Not applicable - I have never been tested positive for COVID-19

4. Do you have a family member in the same household who has tested positive for COVID-19?

a. Yes

b. No

5. For each of the following statements and/or questions, please circle the point on the scale that you feel is most appropriate for describing yourself.

a. In general, I consider myself: 1 (not a very happy person) - 7 (a very happy person)

b. Compared with most of my peers, I consider myself: 1 (less happy) - 7 (more happy)

c. Some people are generally very happy. They enjoy life regardless of what is going on, getting the most out of everything. To what extent does this characterization describe you?: 1 (not at all) - 7 (a great deal)

d. Some people are generally not very happy. Although they are not depressed, they never seem as happy as they might be. To what extent does this characterization describe you?: 1 (not at all) - 7 (a great deal)

6. Over the last two weeks, how often have you been bothered by the following problems?

(Not at all, Several days, More than half days, Nearly every day)

a. Feeling nervous, anxious, or on edge

b. Not being able to stop or control worrying

c. Worrying too much about different things

d. Trouble relaxing

e. Being so restless that it is hard to sit still

f. Becoming easily annoyed or irritable

g. Feeling afraid, as if something awful might happen

7. About how long has it been since you last visited a dentist? Include all types of dentists, such as orthodontists, oral surgeons, other dental specialists, and dental hygienists.

a. 6 months or less

b. More than 6 months, but not more than 1 year ago

c. More than 1 year, but not more than 2 years ago

d. More than 2 years, but not more than 3 years ago

e. More than 3 years, but not more than 5 years ago

f. More than 5 years ago

g. Never have been

8. What was the main reason you last visited the dentist?

a. Went in on own for checkup, examination, or cleaning

b. Was called in by the dentist for checkup, examination, or cleaning

c. Something was wrong, bothering, or hurting (me/SP)

d. Went for treatment of a condition that the dentist discovered at an earlier checkup or examination

e. Other

9. During the past 12 months, was there a time when you needed dental care but could not get it at that time?

a. Yes

b. No

10. What were the reasons that you could not get the dental care you needed?

a. Could not afford the cost

b. Did not want to spend the money insurance did not cover

c. Recommended procedures.

d. Dental office is too far away

e. Dental office is not open at convenient times

f. Another dentist recommended not doing it

g. Afraid or do not like dentists

h. Unable to take time off from work

i. Too busy

j. I did not think anything serious was wrong/expected dental problems to go away

k. Other

l. Not applicable (I could get needed dental care)

11. In general, you would say your health is ____________ (Please pick one)

a. Excellent

b. Very good

c. Good

d. Fair

e. Poor

12. Is today your first time visiting the Maliheh Free Clinic?

a. Yes

b. No

13. How long have you been a patient of the Maliheh Free Clinic?

a. Less than 2 years

b. 2 years or longer

14. How old are you? ____________

15. What is your gender?

a. Male

b. Female

c. Other

16. Were you born in the U.S.?

a. Yes

b. No

17. What country were you born in? (if you were born outside of the U.S.)

_______________________________________________

18. How many years have you lived in the U.S.? ____________ years

19. Which do you consider yourself? (Please pick all that apply)

a. Asian/Pacific Islander

b. Caucasian/Non-Hispanic

c. Hispanic or Latino/Latina

d. African or African American

e. American Indian/Alaska Native

f. Other, please specify: ______________________________________

20. Please describe your highest level of school or degree completed:

a. Less than high school graduate

b. High school graduate or GED

c. Some college

d. 4-year college graduate

e. Graduate school degree

21. What is your current working situation? (Please pick all that apply)

a. Working full-time

b. Working part-time

c. Student

d. Looking for work/unemployed

e. Retired

f. Other, please specify working situation: ______________________________

22. What is your current marital or relationship status?

a. Married

b. Living with a partner in a marriage-like relationship or in a relationship

c. Separated

d. Divorced

e. Widowed

f. Single (never married)

g. Other, please specify ____________________________
